# Mobile Health in Chronic Disease Management and Patient Empowerment: Exploratory Qualitative Investigation Into Patient-Physician Consultations

**DOI:** 10.2196/26991

**Published:** 2021-06-15

**Authors:** Kathrine Stampe, Sharon Kishik, Sune Dueholm Müller

**Affiliations:** 1 Department of Management School of Business and Social Sciences Aarhus University Aarhus V Denmark

**Keywords:** compliance, empowerment, mHealth, patient-physician consultation, power

## Abstract

**Background:**

Chronic diseases often present severe consequences for those affected. The management and treatment of chronic diseases largely depend on patients’ lifestyle choices and how they cope with the disease in their everyday lives. Accordingly, the ability of patients to self-manage diseases is a highly relevant topic. In relation to self-management, studies refer to patient empowerment as strengthening patients’ voices and enabling them to assert control over their health and treatment. Mobile health (mHealth) provides cost-efficient means to support self-management and foster empowerment.

**Objective:**

There is a scarcity of research investigating how mHealth affects patient empowerment during patient-physician consultations. The objective of this study is to address this knowledge gap by investigating how mHealth affects consultations and patient empowerment.

**Methods:**

We relied on data from an ethnographic field study of 6 children and adolescents diagnosed with juvenile idiopathic arthritis. We analyzed 6 patient-physician consultations and drew on Michel Foucault’s concepts of power and power technology.

**Results:**

Our results suggest that the use of mHealth constitutes practices that structure the consultations around deviations and noncompliant patient behavior. Our analysis shows how mHealth is used to discipline patients and *correct* their behavior. We argue that the use of mHealth during consultations may unintentionally lead to relevant aspects of patients’ lives related to the disease being ignored; thus, inadvertently, patients’ voices may be silenced.

**Conclusions:**

Our results show that concrete uses of mHealth may conflict with extant literature on empowerment, which emphasizes the importance of strengthening the patients’ voices and enabling patients to take more control of their health and treatment. We contribute to the state-of-the-art knowledge by showing that the use of mHealth may have unintended consequences that do not lead to empowerment. Our analysis underscores the need for further research to investigate how mHealth impacts patient empowerment during consultations.

## Introduction

### Background

Chronic diseases often have severe consequences for those affected. In addition to the reduced quality of life, chronic diseases may lead to health emergencies, serious complications, and even death [1]. The management of chronic diseases is among the many costly challenges faced by the health care sector [2]. Each year, chronic diseases account for 71% of all deaths globally, prompting the World Health Organization to call for immediate action [3]. Furthermore, chronic diseases contribute to inequality, as they disproportionally affect socially disadvantaged people and impede poverty reduction initiatives [3]. Hence, improving the health of patients with chronic diseases not only improves their quality of life but also eases economic burdens at a societal level. The management and treatment of chronic diseases depend on their impact on patients’ everyday lives [4,5]. Improving patients’ abilities to self-manage their health and make informed decisions is recognized as the key to manage chronic disease at a societal level. This is reflected in numerous national health care strategies that focus on personalized medicine and self-management of disease [6,7]. Self-management refers to patients’ active engagement in the responsibility for their health [1]. In relation to self-management, studies refer to patient empowerment as enabling patients to more meaningfully engage with and assert control over their health and treatment of diseases [8-10]. To support self-management and foster empowerment, mobile health (mHealth) is a cost-efficient method [11].

mHealth is the delivery of health services and information over a mobile or wireless platform [12]. The literature on mHealth in the chronic disease self-management describes various technologies that have been introduced in health care to support self-management and foster patient empowerment [13]. Among these are mobile apps that allow for the collection, management, and sharing of patient-reported outcome (PRO) data. PRO data are direct responses from the patients’ perspectives regarding their health condition, without interpretation from a provider or caregiver [14,15]. Traditionally, PRO data have been collected during physical consultations. However, consultations are often months apart, resulting in recall bias and unreliable data. mHealth may offer a solution by allowing continuous patient input and consequently more reliable PRO data [16]. During patient-physician consultations, PRO data support physicians in evaluating the health status of patients. However, despite studies of how PRO data and mHealth support patient empowerment [17-19], there is a scarcity of research on how mHealth and other technologies are used during patient-physician consultations, and extant studies often ignore the fact that empowerment depends on the patient-physician relationship [8,20]. Consequently, there is limited knowledge of how and to what extent the use of mHealth during consultations affects empowerment. This study addresses this knowledge gap through an exploratory investigation of consultations between children and adolescents diagnosed with juvenile idiopathic arthritis (JIA) and their physician. During these consultations, an app named *How-R-you* supports decision making and patient-physician communication. For analysis purposes, we draw on a theoretical framework based on Michel Foucault’s concepts of power and power technology. Using this framework, we show how the exercise of power is embedded in the different uses of mHealth that constitute specific practices during consultations. In our investigation, we were guided by the following research question:

How is mHealth technology used during patient-physician consultations and to what extent does it support patient empowerment?

The remainder of this paper is organized as follows. First, we account for state-of-the-art knowledge of mHealth in chronic disease self-management and the role of patient empowerment. Second, we present the theoretical framework. Third, we introduce the How-R-you case study and account for our approach to data collection and analysis. Finally, we present our results and discuss their contributions and implications.

### Background Literature

The literature on mHealth in chronic disease self-management covers various technologies and platforms. Technologies include wearables [21], social media platforms [22], and mobile apps [23]. Technologies are studied mainly from one of two perspectives: health care professionals and patients.

From the perspective of a health care professional, mHealth technology provides health care services over geographical distances and supports patient self-management. mHealth may be used to transmit patient data to health care professionals [24] and provide health services in remote areas [25]. Paré et al [24] reported positive effects such as a decreased need for emergency care. Studies also point to greater empowerment, but results regarding clinical outcomes and costs are inconclusive [26].

From a patient perspective, mHealth technology provides many benefits because of the widespread use of mobile phones and the possibility of integrating data from various wearable devices [27]. Patients can monitor symptoms and health issues, which may help them achieve their health objectives [28]. Moreover, various technologies serve as external memories that help patients in remembering health details [29]. This, in turn, supports patients in self-managing their health and coping with diseases [2]. Furthermore, mHealth enables information sharing between patients and health care professionals [19]. As a result, patients increase their knowledge, which helps them to self-manage chronic diseases [19,23]. However, studies have reported problems such as constantly reminding patients that they are chronically ill [30]. Moreover, patients often avoid using technologies because their values and needs are ignored in the design of mHealth [1]. The solution is argued to be a patient-centered technology that strengthens the patients’ voices [1]. In the literature, this is often referred to as a technology that supports patient empowerment.

### The Role of Patient Empowerment

Empowerment implies that patients can fulfill their needs, assert control of their treatment, and independently—or at the very least as an equal partner—decide on appropriate behavior and treatment [10,31,32]. In a widely recognized perspective on empowerment, patients’ subjective experiences of the disease are central to follow-up and treatment. By strengthening patients’ voices, empowerment is an engagement of patients that goes beyond mere compliance [33]. However, in much of the literature, empowerment is also conceptualized as the measurable result of an intervention, and the role of mHealth technology in empowering patients is thus paradoxically referred to, discussed, and measured in terms of compliance with established norms and treatment [34]. For instance, Lasorsa et al [2] designed an mHealth technology to support patients’ self-management and everyday decision making. The solution is said to incorporate empowering features and a means to ensure compliance with medical treatment. Similarly, Fioravanti et al [35] studied an mHealth strategy aiming to improve medical compliance through empowerment. This strategy promotes lifestyle changes and improves compliance. With such studies, the literature articulates a paradoxical conceptualization of empowerment. Patients gain control in the sense that they improve their ability to comply with the physicians’ medical advice. Following this logic, empowerment is less about patients’ everyday lives and their subjective experiences with the disease than their compliance with treatment plans. According to this conceptualization, empowerment is disease- rather than patient-centered, and technologies continue to privilege a clinical perspective [33].

Empowerment must be patient-centered if the patients’ voices are to be truly strengthened. Dadgar and Joshi [1] argue that mHealth must cater to patients’ values to affect empowerment. Butterworth et al [36] combined patient and clinical perspectives to create a technology-enabled education system that, on one hand, serves patients’ needs by improving their ability to make informed decisions and, on the other hand, supports health care professionals by teaching patients to care for themselves and reminding them of appointments. Storni [33] proposed a patient-centered mHealth journaling tool that considers patients’ individual needs and enables self-monitoring personalization. Technologies offering privilege to the health care professional’s perspective assume that health care outcomes are always clinically measurable and necessitate compliance: “Such a strategy may verge on the paradox of ‘empowering’ patients to better silence their voices, but poorly supports them with the practicalities and complexities of dealing with the disease” [33]. Patients experience diseases subjectively, and thus should not be reduced to generic medical conditions [33]. Otherwise, patients’ insights and knowledge are neglected at the risk of ignoring relevant aspects of their lives with the diseases. This is particularly important as patients’ lifestyle choices and their everyday lives are key to managing chronic diseases [5]; hence, the emphasis on empowerment as strengthening patients’ voices and enabling patients to take control of their health and treatment.

Although the referenced studies constitute important contribution to the literature on digitally enabled chronic disease self-management, the extant literature does not investigate the role of mHealth technology during patient-physician consultations, and studies thus tend to ignore that empowerment is dependent on the patient-physician relationship [20]. Consequently, the state-of-the-art knowledge of how to use mHealth technology and PRO data during consultations, and whether they promote patient empowerment, is insufficient. Use of mHealth that reduces patients to generic medical conditions is not conducive to empowerment and may result in patient-physician miscommunication [37]. Therefore, research is needed to investigate how mHealth is used and how it affects empowerment. This paper addresses this knowledge gap and analyzes the use of mHealth technology during patient-physician consultations.

## Methods

### Theoretical Framework

We analyzed the use of mHealth through a Foucauldian perspective. Foucault’s concept of power as embedded in institutional practices and exercised over free subjects is particularly useful while trying to understand how power is exercised in modern state institutions (eg, hospitals) and investigating the aforementioned tensions between compliance and empowerment [38,39].

Foucault’s understanding of power stands in stark contrast with the conceptualization of power as an oppressive force [40]. Instead, power is productive as it creates subjects with specific characteristics and attributes. Moreover, power cannot be possessed, and its locus cannot be pinpointed with reference to a person in a leading position. Power *is* only insofar as it is exercised, meaning it emerges only in relationships, “...as a mode of action upon the actions of others” [40]. Thus, it presupposes the ability of subjects to act freely. Therefore, the study of power must be conducted as an analysis of the relational practices through which some are directed to adjust their behavior according to certain norms [40]. An example of such an analysis is found in “Discipline and Punish” [41]. Here, Foucault analyzes discipline as a form of power that gains ground in the 19th century, and which—as we will show—is being continuously refined today. His analysis takes as its objects the various practices in prisons, through which the inmates are governed to discipline and adjust their behavior. Discipline is a power that rectifies norm-deviant behavior by initiating normalizing processes. By the same act, it creates productive yet docile subjects [41].

For the purposes of our analysis of mHealth use during patient-physician consultations, Foucault’s concept of power technology provides a potent analytical tool. Power technology refers to something that is mobilized in specific contexts to govern the conduct of others. Power technology structures and organizes contexts in specific ways. It may render some things visible, whereas others are left invisible or hidden [42]. Nothing is a power technology in and of itself. It comes to constitute a power technology, only when, in a certain context, it is invested with a specific rationality and used to govern the conduct of others [43]. This does not mean, however, that mHealth *is* inherently a power technology. Rather, construing it as a power technology enables us to analyze how mHealth organizes consultations and is used to govern behavior. Only as far as it organizes consultations in particular ways, mHealth is constituted as a power technology.

In this study, we drew on Foucault’s concepts of power and power technology to analyze the different uses of the How-R-you app that constitute specific practices during consultations. Thus, although other frameworks conceive power as something that can be possessed and used for different purposes, the Foucauldian perspective allows us to analyze how power emerges subtly through health care practices. We may thereby gain new insights into how technology, which is seemingly empowering patients, also governs their behavior in specific ways.

### mHealth as a Power Technology

The propagation of mHealth and other technologies, meant to engage and empower patients, must be viewed in light of the historical development within health care, with an increased focus on patient-centered care. It has been argued that we have seen a crisis in traditional management and authority, and that physicians must increasingly refrain from exercising authoritative, paternalistic power. Instead, the patient-physician relationship must be based on dialogue [42]. Especially with the approach often taken to chronic diseases, where patients’ lifestyle choices are in focus, the physician must incorporate the patient’s perspective, on account of the premise that any real lifestyle changes come from within the patient [42,44]. This creates challenges in the management of patients with chronic diseases: health care must be provided without commanding or ordering patients.

In this paper, we argue that mHealth is sometimes used as a solution to this challenge. mHealth technologies invoke a seemingly unoppressive power because they provide patients the right to speak and be heard, which is perceived as liberating and empowering [42]. They give patients a voice; yet, they are a means to ensure compliance. Specific practices during consultations highlight this point. Thus, we argue that mHealth does not simply liberate patients from being subjugated to power; rather, it reinstalls patients in new power relations, in which their seemingly empowered voice is used to govern them in specific directions. Thus, mHealth governs by ensuring that the patients are, in Rose’s terms, “bound into the language of expertise at the very moment they are assured of their freedom and autonomy” [45]. In other words, the patients are paradoxically governed exactly through the freedom that mHealth seems to provide them. In the following analysis, we argue that How-R-you is constituted as a power technology. Although it seems liberating, it also organizes consultations to ensure patient compliance according to medical recommendations. Thus, it is invested with a governmental rationale.

### How-R-you

**Figure 1:**
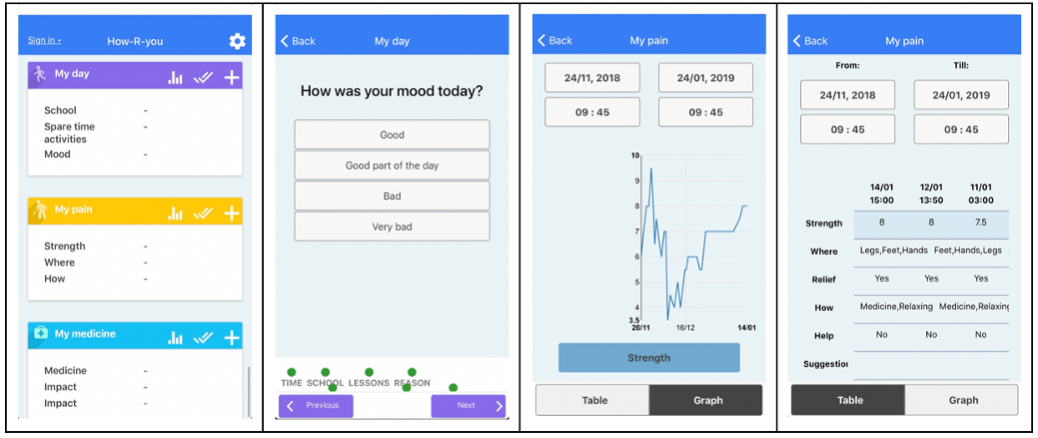
How-R-you modules, question, graph, and table examples.

How-R-you was chosen for this study because it was developed with a focus on empowerment and strengthening of patients’ voices. The app was developed by Business Academy Aarhus with input from patients, health care professionals from Aarhus University Hospital, and researchers from Aarhus University. The app was commissioned by physicians to gain a better understanding of patients’ everyday lives with chronic diseases and the symptoms they experience. How-R-you allows patients to continuously report on their health and monitor their well-being using PRO data. Specifically, the app enables 4 of 7 self-management activities [46] in patients’ everyday life (drug management, communication with health care professionals, social support, and symptom management) by allowing patients to track drug use and monitor symptoms, and by supporting communication with both health care professionals and other stakeholders, especially parents [47]. How-R-you is organized into modules that contain health questions. The modules are “My medicine,” “My pain,” “My day,” and “My night” (Figure 1).

Modules and questions were developed and revised iteratively, and both patients’ and physicians’ needs were included in the design. The modules are configurable, which enables personalization according to the specific preferences of patients and physicians. Furthermore, How-R-you allows patients and physicians to gain an overview of historical data by converting them into graphs and tables.

### Data Collection

We relied on data from a 2-year ethnographic field study of the impact of digital technology use in everyday life with JIA. The ethnographic approach helped us understand real-world problems empirically before seeking to explain them theoretically [48-50]. The fieldwork yielded both overall and individual accounts of 6 patients’ everyday lives with JIA and their visits to the hospital. These accounts constituted thick descriptions [51] of patients’ information needs and the extent to which How-R-you as an mHealth technology created value for patients [47]. The fieldwork comprised participant observations and interviews. Following Spradley [52], observations are always participatory, but to varying degrees. Observations were descriptive at the outset of the study to gain insights into the sociotechnical aspects of technology use. The researchers participated passively at the outset. Subsequently, observations became more focused, and participation became more involved in the sense of taking part in patients’ daily activities. Thus, additional data were collected to gain a deeper understanding of selective aspects, that is, how How-R-you is used during consultations. The data were collected by the second author (KS), with support from the third author (SDM) who participated in the hospital. For this study, we carefully selected 6 specific observations of consultations as the empirical basis for our analysis. All observations were carefully planned and subsequently discussed to ensure reliable and valid interpretations.

The 6 cases comprised 1 consultation with each of the 6 patients included in the ethnographic study [47]. Patients were selected based on four criteria. First, all patients had the same disease (ie, JIA). Second, they were not diagnosed with other diseases (comorbidities). Third, both female and male patients were included. Finally, 1 female and 1 male patient were selected to represent each of the three predetermined maturity groups (7-10, 11-13, and 14-17 years old) that were a part of the study. The age groups were based on literature describing the medical assessments of JIA children and adolescents’ cognitive and physiological development [53]. Children can assess their pain using a visual analogue scale, as in the case of How-R-you, from age 6 [54,55]. Furthermore, patients aged 18 years and above were treated as adults at Aarhus University Hospital. This means that our target group was 6-17 years old. In addition, the literature divides JIA children into 2 age groups; however, the cutoff age varies [53] between 12 [56] and 13 years [57] (Table 1).

**Table 1:** Overview of patients.

Patient	Sex	Age (years)
Nina	Female	7
Robert	Male	10
Rachel	Female	12
Oliver	Male	12
John	Male	14
Sophie	Female	16

We specifically selected these 6 consultations because they were held during the final stages of the fieldwork. During this stage of the fieldwork, both patients and the physician had become relatively familiar with How-R-you, and, therefore, technical questions and issues were less frequent. Moreover, at this selective stage of the fieldwork, we relied on extensive experience after 2 years of descriptive and focused interviews and observations of the patients’ lives. During this time, the app underwent iterative development because of feedback from patients and physicians. Consequently, at this stage, we focused our observations specifically on the actual use of How-R-you during consultations [47]. Thus, these 6 consultations provide an ideal basis for our investigation into how How-R-you is used during consultations and how it affects empowerment.

All consultations were performed by the same physician to limit in- and cross-case variations. Although all the children’s parents were present at the consultations, their involvement varied across the age groups. Parents of young children were highly involved, whereas parents of adolescents were not involved [47]. The duration of consultations varied between 20 and 30 minutes depending on the treatment needs and issues raised during conversation with the physician. The consultations were documented through participant observation [52], audio-recorded, and transcribed. Transcriptions were performed by the first (SK) and second (KS) authors. The consultations were discussed in detail to ensure that all aspects were included in the analysis. The observations were carefully planned with both the physician and patient. In accordance with the nature of passive participation, we interfered as little as possible in the consultations and only interacted with participants when encouraged by patients or the physician. To minimize the disruptive effects of our presence, we took field notes with pen and paper from the corner of the consultation room. Field notes were available to the participants.

### Ethics

All patients, parents (due to the patients’ ages), and the physician gave informed consent to all parts of the fieldwork. The physician collaborated with the research team throughout the fieldwork and participated in discussions about ethics. The patients and their parents were invited to a private informal meeting with one of the authors, who explained the details of the research projects. At least one week later, the author called patients and parents, who had expressed an interest in joining the study at the meeting. This gave patients and parents an opportunity to reconsider their interests and ask questions about the research project. Consent was given at the end of this conversation and more specific permissions, such as audio recording and taking pictures, were obtained throughout the entire study. The study complies with the ethical principles for medical research involving human subjects and with the American Anthropological Association’s ethical principles for ethnographic fieldwork [58]. The study did not require approval from an ethical review committee according to the Danish National Committee on Biomedical Research Ethics.

### Data Analysis

To address the research question, we draw on Foucault’s concepts of power and power technology in an embedded case study of patient-physician consultations. This means that we seek to understand how How-R-you is used during consultations, what kind of practices it constitutes, and the rationale that drives the organization of these practices.

In our analysis, we are not concerned with the physician’s intentions and motivations behind the use of the app, but only with how it is used and what it does to the consultation and the patient-physician relationship. Our interest lies not in the participants but in how the practices themselves lay the foundation for the exercise of power by organizing the consultation and inducing a certain behavior. Thus, How-R-you is not used by someone *in* power to assert power. Rather, power emerges as the way in which How-R-you organizes the relationship between the patient and the physician (Textbox 1).

**Data coding.**

**Identification of Deviation**

- Pain
- Disturbed sleep
- Bad mood
- Physical inactivity

**Structuring of Conversation**

- Specifics about the deviation
- Patterns between disease indicators
- Ways of handling the deviation

**Discipline**

- Education
- Commending
- Questioning

Consultations were analyzed through an iterative process. By observing the consultations and listening to the recordings numerous times, we gained a preliminary understanding of the practices. Next, we transcribed and coded the data using the qualitative data analysis software NVivo 12 (QSR International). Top-level codes were identified based on our preliminary understanding of practices and our theoretical framework. This means that we sought to understand how the app is used organizes certain practices in specific ways and how it is invested with a specific rationale. Thus, we asked the following questions: How is the app used in specific practices? What registrations become visible to the physician and what are left unmentioned? How are the registrations used in conversation? Which topics become important during the conversation, and which are rendered irrelevant?

All authors were involved in data coding and analysis. The first (SK) and second (KS) authors provided deep insights and domain knowledge from participant observations and interviews. The third author (SDM) contributed to the in-depth text analysis of communication content, check coding, and challenging the interpretations of other authors. Through this process, we meticulously went through the transcriptions and coded the data according to the top-level codes. This led to the identification of sublevel codes for each top-level code. The process provided an in-depth understanding of the consultations and practices described in the following analysis.

## Results

### Overview

Our analysis reveals three interrelated practices around the use of How-R-you: ordering the patients’ words; identifying deviations and structuring of patients’ speech; and disciplining of patients. In the following, we argue that these practices collectively constitute How-R-you as a power technology. We devote each of the following three subsections to each specific practice. Finally, we conclude this chapter by summarizing the results.

### Ordering the Patients’ Words

How-R-you allows patients to register and monitor their health data. These data were examined by the physician and included in the health assessments of the patients during consultations. Therefore, How-R-you is believed to strengthen patients’ voices by giving them the ability to provide personal insights. Before the introduction of How-R-you, patients arrived at consultations without a record of their health and well-being since their last consultations. In fact, patients and parents (who support their children) rarely take notes on their health from day to day. The result is recall bias when asked to account for their well-being since the last consultation. In How-R-you, patients make continuous health registrations by answering specific questions in the app. Thus, How-R-you constructs and builds a record of PRO data. During consultations, the data were presented to the physician as the patients’ own words. However, in How-R-you, the registrations are ordered in certain ways, for example, in graphs that show how the patient felt each day since his or her last consultation:

Then we can use the registrations you have made (...) we just need to construct the graphs, because, you know, that’s what’s important—how your day has been.

Physician

The technology itself becomes an actor with a voice of its own. It simplifies patient experiences and confines registrations to specific questions. For instance, moods and feelings, which may be difficult to explain, are registered using smiley and slider scales (Figure 1). These registrations are presented in tables and graphs that allow physicians to examine the data. As a result of this ordering of the patients’ words, physicians can quickly sum up otherwise complex experiences. For example, in the following quotation, a patient’s mood and physical activity since the last consultation are summarized as follows:

Well, your mood seems to have been okay despite some tiresome nights. And your level of activity has been normal.

Physician

Thus, How-R-you is not a neutral instrument. It allows patients to continuously register health observations by answering specific questions, and it presents these registrations in a particular manner. The way How-R-you is used constitutes a practice that orders patients’ words but presents them as their own voice. This allows for a seemingly empowering process through which patients’ insights are incorporated during consultations. However, the ordering of the patients’ words only enables very specific observations of the patients’ lives with the disease. In the following section, we expand on this last point.

### Identification of Deviations and Structuring of Patients’ Speech

The ordering of patients’ words exerts a structuring effect on conversations and patients’ speech during consultations. During the physician’s examination of data, it is evident that the physician scans the app modules for deviations, such as pain, disturbed sleep, mood swings, or physical inactivity. In other words, How-R-you renders deviations visible to physicians. How-R-you structures the conversation around the observed deviations. It does not reduce conversation as it expands it around very specific topics. With reference to their registrations in How-R-you, the patients were encouraged to speak and describe the deviations. Their speech is therefore structured by limiting the conversation to (1) specifics regarding the deviation, (2) patterns across disease indicators, and (3) how they *handle* the deviation:

What should I look at...your pain? Do you ever feel pain? Yes, you do actually—pain in the legs.

Physician

The above quote is from Nina’s consultation, where the physician browsed the “My pain” module. The physician observed that Nina registered pain in her legs. The conversation then unfolds around this deviation. The physician probed the specifics of the pain, and Nina’s mother described when the pain usually occurs and what they did to relieve it. In other words, it is about how they *handle* the deviation. This conversation helped the physician ascertain the cause of the pain.

In Robert’s case, the physician’s data analysis also revealed pain, and the conversation again focused on this topic. Neither Robert nor his father could remember the specific incidents where Robert experienced pain. However, Robert’s father was uncertain whether the pain is attributable to arthritis or because Robert engaged in new and intense physical activities. This indicates that pain is not only related to arthritis:

...when it is something [pain] with the hand, well, that sounds like it’s because you do something that you regularly don’t, right?

Physician

During Rachel’s consultation, the physician observed that Rachel had registered mood swings. However, Rachel attributed the cause to “...something at school,” which seemed to indicate the physician that her mood was not relevant to the consultation. The physician continued by looking at other data in How-R-you and suggested that mood swings are connected to another disease indicator, namely physical inactivity. Similarly, Sophie attributed her mood swings to the lack of energy caused by a combination of pain and stress at work. In this case, the physician ascertained through How-R-you that there was no correlation with other disease indicators. The physician then told her that to cope with these mood swings, she will have to figure out what she can and cannot do:

That’s part of what’s going to be your challenge—to figure out the balancing act in what you are able to do and what you cannot do.

Physician

In the examples above, the structuring of conversations around deviations and the patients’ own accounts led the physician to draw conclusions. The examples show that deviations are well-handled (Nina), do not relate directly to the arthritis (Robert and Sophie), or seem not relevant to the consultation (Rachel).

Notably, although the patients’ perspectives have been included in the design of How-R-you to foster empowerment and strengthen the patients’ voices, the app seems to structure the patient-physician consultations in a manner that seems to provoke somewhat opposite effects. One may argue that during the consultations, How-R-you renders certain relevant aspects of patients’ lives irrelevant. These are aspects that the patients themselves bring up or have registered in the app (eg, what happens at school), indicating that they are relevant to their lives and, thus, to the self-management of their disease. However, the way How-R-you organizes registrations, for example, in graphs and tables, seems to leave these aspects in the blind spot of the physician’s gaze, which is focused on deviations and patterns across disease indicators. Data are shared, but patients’ voices are only selectively included as a basis for disease management and treatment during consultations. This is because How-R-you only enables very specific observations of patients’ lives with the disease. As our analysis suggests, these observations leave relevant aspects unnoticed.

In other cases, patients’ speech becomes a reference point for the exercise of power and identified deviations become targets for corrective, disciplinary power. We provide examples in the following section.

### Disciplining of Patients

Our argument in the following is that by facilitating, ordering, and structuring patients’ words and speech, How-R-you allows for the exercise of disciplinary power over patients with reference to their own spoken truths. The physician avoids referring to her superior medical knowledge and position. Instead, by referring to patients’ own registrations, disciplining is conducted without the exercise of oppressive power. Specifically, disciplining takes the form of education, commending “right” (read in a medical sense) behavior and questioning patients.

During John’s consultation, the physician browsed the How-R-you registrations and observed a deviation regarding his sleep patterns:

This is your sleep. So, disturbed [sleep], then a little steady and then very [disturbed].

Physician

John was asked to account for details around his sleep and he responded by describing his tiredness. This deviation, observed through the app, and the patient’s description form the background for the physician’s comment:

...it’s still two days, where your sleep was very disturbed, so that’s something, where you should keep in mind that the best thing that sleep knows is consistency. So, you go to bed at the same time, you wake up at the same time, you don’t change it too much in the weekends...

Physician

With reference to the deviation, educating the patient about how to improve his sleep is legitimized. In other words, education is necessary because “it’s still two days, where your sleep was very disturbed.”

The physician continues by describing optimal bedroom temperatures and warnings against the effects of technology use before sleep:

This means that all kinds of tablet, tv, computer, and all that stuff should preferably not be switched on [...] So I’ve just asked you to use an app [How-R-you], and now I’m asking you to really not use it too much before bedtime, or at least that’s something to think about.

Physician

Thus, education and suggested behavioral changes are used to *correct* the deviation. Both John and his mother engaged in conversations with the physician and showed signs of compliance. John mentioned that he switches his iPhone lighting to *night shift*, which changes the display to warmer colors. His mother emphasized that they imposed stricter bedtime rules regarding when to turn off video games. In response, the physician replied: “Right, it’s those video games.” Thus, John and his mother show compliance by explaining how they are going to *correct* the deviant behavior. A part of disciplining the patient is to make him or her recognize the need for behavioral change. This involves a conversation about the deviation during which compliant behavior is constructed as *right* and noncompliant behavior (eg, playing games before bedtime) is articulated as *wrong*.

During Rachel’s consultation, the topic of physical inactivity emerged because she had registered periods of pain. The physician noted that the number of days of pain correlated with days where she was less active. This topic was revisited later during consultation. Although the physician did not reference the observed deviation in the subsequent disciplining, there was a correspondence between the observed deviation (physical inactivity) in How-R-you and the discipline. Thus, the registrations in How-R-you reveal that pain may be caused by physical inactivity (the deviation). In this sense, the registrations form the background for the following conversation:

Yesterday, I walked 30,000 [steps], because I rode [my horse] twice, then I had physical education and rode my bike and stuff.

Rachel

Well-done [...] I thought you’d be at around 8,000-10,000 [steps] most of the times, unless you’re one of those, who play Fortnite, right.

Physician

Right.

Rachel

Then it’s at a zero.

Physician

Rachel’s response revealed compliant behavior (physical activity). This behavior was encouraged by the physician who commended her while at the same time alluded to noncompliant (ie, *wrong*) behavior, namely inactivity and too much gaming. Here, discipline takes the form of commending compliant behavior and constructing noncompliant behavior as *wrong*.

During Oliver’s consultation, the physician observed that Oliver had registered periods of pain. When asked about the specifics, he revealed deviant behavior in the form of physical inactivity:

So, you nevertheless had one day in January, where you felt pain in your leg.

Physician

Yes, so I had a 7.5 (on the pain intensity scale) [...]

Oliver

Was it related to football, or was it just kind of sudden?

Physician

No, I don’t really play football, just in the breaks...[Oliver]

The physician returned to this issue later during the consultation:

Then what about getting in better shape. Besides playing a bit of football in the schoolyard, is there something specific you do?

Physician

Well, I actually think I’m in OK shape.

Oliver

Right OK, but I think we’ve talked about it a couple of times.

Physician

By referring to the patient’s own statement, the physician can ask the question:

Then what about getting in better shape [...] is there something specific you do?

Hence, the physician tries to *correct* the deviation, that is, encourage the patient to improve his physical condition. This type of questioning continues and included the nurse who asked whether the patient participates in physical education. Oliver’s mother showed compliance and mentioned that they have talked about taking long walks during weekends and signing up for membership at a fitness center. The physician commented “Well, it sounds like you have a lot of good things up and running.” Again, compliant behavior was encouraged.

### How-R-you as a Power Technology

Our findings suggest that three interdependent practices constitute How-R-you as a power technology: first, it orders patients’ words and presents them in a specific way. Second, it structures the patients’ speech during conversations around deviations. In doing so, it may have a silencing effect on patients’ voices. Third, it allows for the disciplining of patients by referring to their own words and speech, thereby enabling an exercise of power that does not appear oppressive. Thus, these practices are means by which disciplinary power is exercised, which entices, motivates, and commends the compliant behavior, and at the same time constructs noncompliant behavior as *wrong*. Naturally, we do not argue that specific suggestions in the discipline of patients are wrong or damaging. However, we argue that compliant and deviant behaviors are determined based on medical knowledge and expertise, and patients’ voices are not included in this assessment. In Foucauldian terms, the exercise of power might produce more healthy subjects observed through a medical gaze focused on lifestyle choices, but in doing so, it might ignore relevant aspects of the patients’ lives. This conflicts with the purpose of empowerment in chronic disease self-management, which emphasizes the strengthening of patients’ voices and the importance of patients’ everyday lives with the disease. As a power technology, How-R-you is an efficient management tool that spares physicians from lengthy conversations, which are often affected by patients’ recall bias. Instead, it presents PRO data in a structured manner that allows physicians to quickly identify deviations. In a Foucauldian sense, the deviations become the object of a power that *corrects* behavior. It is therefore questionable whether How-R-you supports patient empowerment during consultations. Our analysis revealed practices in which patients’ behaviors were governed toward specific ends, which might result in compliance. However, as a result, their voices may not be heard, and thus, relevant aspects of their lives may be ignored. In the following section, we discuss the implications of these results.

## Discussion

### Contribution and Implications

First, our results are surprising because they are in conflict with the perceived purpose of mHealth technology as described in the literature, which defines empowerment as giving patients greater control of their health and disease treatment [8,31,32]. Moreover, the findings are particularly interesting because they reveal a dimension to the study and use of mHealth technology, which has so far been largely neglected by the extant literature. Studies emphasize the importance of including patients in technology design [1,33,36] and studying the patient-physician relationship through data collected before, between, and after consultations [19]. Although How-R-you is developed through a user-centered design process, our study shows that the use of technology constitutes practices that promote compliance. This means that although user-centered design is a prerequisite for empowerment, it is not sufficient. The actual use of technologies during consultations also affects empowerment. In this particular case study, there is evidence to suggest that the technology could have a disciplinary rather than empowering effect during consultations, as it specifically structures the consultation around deviations and compliance. Our qualitative research design, with its focus on consultations from a process perspective, allows us to gain insights into how a particular technology orders and structures the consultation and patient-physician relationship and what the implications are for patient empowerment. Thus, we contribute to state-of-the-art knowledge by showing that technology use may have negative effects on empowerment, and we underscore the importance of future research to investigate the actual uses of different technologies.

From the perspective of the physician, our results show how technologies such as How-R-you construct records of patient registrations of PRO data, which are subsequently used to structure conversations with patients and discipline them by referring to their own words and thus foster compliance. This discipline occurs through education, commending compliant behavior, and questioning deviations. The disciplining of patients may also take the form of, for example, repeating patients’ own words that indicate a desire to change behavior toward compliance [42]. Disciplining in this sense is about making use of patients’ own words and statements to make them recognize a willingness to comply with treatment plans and medical recommendations. Physicians may use technologies during consultations to improve medical compliance, and our results show the manner in which this discipline occurs. Physicians may also use our study to increase their awareness of how technologies structure their observations. In doing so, they may come to appreciate that information other than that which the technology presents as *deviant* may also be relevant to the patients’ lives and thus for their ability to self-manage their disease.

By contrast, to assert control, patients need to become aware of practices reducing them to mere deviations that must be *corrected*. Thereby, patients can take advantage of the empowering potential of technologies to steer conversations toward registrations that are important to them. For instance, patients could direct attention to registrations that might have gone unnoticed by the physician and expand on these registrations by dwelling on details that are important to them. Thus, as our findings underscore, this empowering potential does not reside innately in technologies. To nurture this potential, both physicians and patients must become aware of the way in which technologies organize and structure their relationships. Practical means of doing so include physicians spending time examining the registrations with the patients. In doing so, not only should deviations and noncompliance be attended to, but the physician should also initiate dialogue around what may at first glance seem like unremarkable registrations, asking questions such as, “How did you feel during this time, when you were seemingly physically active?” and “Did you do or notice something different?” Thereby, patients are encouraged to speak not only about reasons for deviations or noncompliance but also about other aspects that are relevant to their lives with the disease.

In conclusion, we argue that mHealth technology cannot simply be assumed to be empowering but must be studied in the specific contexts in which it is used. On one hand, it may effectively reduce the recall bias and provide physicians with more data. On the other hand, while studying empowerment from a patient perspective, it cannot be disentangled from its specific uses during consultations. Empowerment depends on patient-physician relationships, where data are shared and where patients’ voices and experiences are heard. This has implications for the design of mHealth technologies. Given that empowerment depends on allowing patients’ voices and their specific experiences to be heard, designers should prioritize the development of features that support dialogue around specific patient experiences. For instance, instead of turning quantitative PRO data into graphs, the technology may allow patients to register experiences for follow-up and elaboration during consultations. Overall, in addition to emphasizing the collection and sharing of data, mHealth technologies could incorporate design features that aim to invoke dialogue between patients and physicians where the patients’ lived experiences with the disease are heard.

### Call for Research

First, this study raises the question of whether other types of mHealth technology used in chronic disease management have similar effects. As mentioned, in extant literature, the question of how empowerment happens (or not) during consultations is understudied. Our study underscores the importance of studying how technology impacts patient-physician consultation to understand its potential to empower patients. We neither intend nor are we able to reveal a general pattern in the use of mHealth technologies during consultations. For generalization, additional cases need to be investigated, including different technologies, practices, and patient groups. Second, research is also needed to understand how the identified practices influence the work practices of physicians. Foucault emphasizes that power is not exercised by subject A over subject B. Power is relational and thus affects both subjects. Technologies that privilege patients’ autonomy challenge physicians’ reliance on medical knowledge. The question is, therefore, how physicians redefine themselves when they are expected to manage without managing? Third, our results raise questions regarding the relationship between empowerment and compliance, which calls for future investigations. Is the former always preferable to the latter, or is it possible to achieve both at the same time without compliance, preventing patients from assuming control and vice versa? Furthermore, it is possible that practices similar to those identified in this study lead to patients feeling empowered despite being disciplined. This raises questions about *real* versus perceived empowerment. To what extent does perceived empowerment improve clinical outcomes, and if it involves disciplining the patients, to what extent does it support patients in dealing with their diseases?

### Limitations

A number of limitations of our study deserve mention. First, in our analysis, we did not differentiate between patient ages. For our analytical purposes, we focused on how How-R-you structures consultations. However, we recognize that physicians may approach consultations with patients from different age groups differently, and that different age groups react differently to mHealth technology and medical advice. Second, as the patients in our study were children and adolescents, the way How-R-you structures the consultations may vary from that of adults, and power relations will take different forms. Although we recognize that consultations with children and adults differ, we believe that the questions concerning mHealth and power relations raised by our study apply to both types of consultations. Thus, while our study does not make these differentiations, it points to the importance of studying the use of mHealth in different contexts and patient groups.

### Conclusions

In this paper, we investigate the use of mHealth technology during patient-physician consultations with a focus on empowerment. We thereby shed light on an area that remains understudied, namely the use of mHealth during consultations. On the basis of our analytical findings, we argue that the use of the How-R-you app constitutes it as a power technology that promotes compliance with medical recommendations. Our findings contribute to the extant literature by showing how practices, emanating from the use of mHealth technologies, may have unintended consequences. This calls for future research into the use of various types of mHealth technology during consultations to investigate whether they in fact support patients in asserting control of their health and disease treatment.

## Abbreviations

JIA: juvenile idiopathic arthritis
mHealth: mobile health
PRO: patient-reported outcome
